# The language of alcohol: Similarities and differences in how drinkers and policymakers frame alcohol consumption

**DOI:** 10.1111/dar.14056

**Published:** 2025-04-10

**Authors:** Emma Moreton, Emma Davies, James Morris, Richard Cooke

**Affiliations:** ^1^ University of Liverpool Liverpool UK; ^2^ Oxford Brookes University Oxford UK; ^3^ London South Bank University London UK; ^4^ University of Staffordshire Stoke‐on‐Trent UK

**Keywords:** alcohol, binge drinking, linguistics, policymakers, young adults

## Abstract

**Introduction:**

The primary objective of the paper was to compare semantic domains reported by drinkers and policymakers in their alcohol consumption narratives. The research question was ‘How do drinkers and policymakers use semantic domains to construct alcohol consumption narratives?’.

**Design:**

Secondary analysis of open‐ended survey responses (The Drinker Corpus: TDC) and three English alcohol policies (The Policy Corpus: TPC).

**Methods:**

Wmatrix software was used to identify semantic domains that appeared more frequently in our corpora compared to general usage. Wmatrix outputs a log‐likelihood (LL) score; a score of 6.63 corresponds to a *p* value of 0.05, indicating frequently used domains.

**Results:**

Five domains appeared more frequently in both corpora than general usage: ‘Cause & Effect/Connection’; ‘Disease’; ‘Drinks and Alcohol’; ‘Excessive drinking’; ‘Knowledge’ (LL >226.68 for all). Domains were represented differently in the two corpora; the TPC focused on long‐term health consequences, like liver disease, whereas the TDC talked about short‐term consequences like hangovers. The ‘Emotional actions’ domain appeared in the TDC more than expected (LL = 231.26). Drinkers reported experiencing positive and negative emotions following drinking. The ‘Social actions/states/processes’ domain was used more frequently in the TPC than expected (LL = 408.17). Policymakers talked about changing ‘behaviour’ in partnership with organisations rather than working with drinkers.

**Discussion and Conclusions:**

This study shows that while drinker and policymaker alcohol consumption narratives draw on the same semantic domains, how these domains are used to construct these narratives differs. To improve the effectiveness of policy initiatives, we recommend greater dialogue between policymakers and drinkers.

## 1 INTRODUCTION

A key goal of alcohol policy is to reduce population‐level alcohol harms. One way to achieve this goal is to propose policy initiatives that reduce alcohol use among at‐risk sub‐groups. Young adults (aged 18–30; [1]) have been identified as an at‐risk sub‐group due to their tendency to engage in binge drinking, that is, consuming more than a threshold number of drinks, or volume of alcohol, during a drinking episode [2]. Binge drinking is associated with an increased risk of experiencing acute alcohol harms including accidents, blackouts, hangover, nausea, unsafe sex and violence [3, 4, 5].

At the start of the 21st century, successive English governments pursued a policy agenda to reduce alcohol harms by targeting young adults' binge drinking [6, 7, 8]. These policies contained measures including labelling alcohol products with information about alcohol units^1^ and warnings to avoid drinking when pregnant, and health campaigns like ‘Know your Limits’ [9] targeting young adults' binge drinking.

Such policy narratives offer one lens through which to view young adults' drinking behaviour—as a problem to address, to increase the health of the population. However, because these narratives encourage a reduction in alcohol use they can challenge drinkers' views about their drinking behaviour and, in turn, their drinking identity, meaning they are often contested. Oldham et al. [10] found interventions to reduce drinking were judged unfavourably if they could interfere with the social gains associated with alcohol use among a sample of higher and increasing risk UK drinkers. Other studies have found UK drinkers challenge the amount of alcohol specified in alcohol guidelines [11] and those who drink more alcohol are more likely to reject alcohol health risk messages [12].

In contrast to the harm focus of policy narratives, young adults' narratives typically recognise the potential for both harms and gains when binge drinking; young adults link binge drinking with social gains such as bonding with others, creating shared memories, relaxing and reducing social anxiety [13, 14]. Such findings map onto psychological theories, such as Alcohol Expectancy Theory [15], the incentive motivation model [16], the Prototype Willingness Model [17] or the Theory of Planned Behaviour [18], which have been used to predict young adults' binge drinking [19, 20]. Common to all models is an acknowledgment that multiple psychological factors contribute to an individual's decision to drink alcohol, or not drink alcohol, and to how much to drink. For example, Cox and Klinger's [16] incentive motivation model identifies four key motives for drinking, including to manage emotions or feelings by enhancing an evening or coping with negative emotions. There is evidence that drinking motives predict young adults' binge drinking [21, 22]. Thus, policy focusing solely on the avoidance of harm will fail to fully encompass psychological factors that inform binge drinking among young adults.

So, the same phenomenon—young adults' binge drinking—can be viewed differently from policy and drinkers' perspectives. This accords with calls to attend to how problems are framed [23], including in the context of alcohol policy [24], particularly in terms of understanding how embedded meanings and power dynamics are important to robust evaluation [25]. We believe it is important to compare how policymakers and young adult drinkers frame binge drinking narratives to identify similarities and differences. How can we compare these narratives? One way is to use linguistic analyses. This approach involves first identifying the words, phrases and grammatical patterns that are frequently used by young adult drinkers versus policymakers and then comparing these findings against a larger corpus of general English usage to test for statistical significance. We take as our starting point the Systemic Functional Linguistics view [26] that language both reflects and constructs reality:

‘In this view language does not passively reflect reality; language actively creates reality. It is the grammar – but now in the sense of lexicogrammar, the grammar plus the vocabulary, with no real distinction between the two – that shapes experience and transforms our perceptions into meanings. The categories and concepts of our material existence are not “given” to us prior to their expression in language. Rather, they are construed by language, at the intersection of the material with the symbolic.’ [16, p. 145].

The main aim of the present paper was to use linguistic techniques to offer a novel lens through which to see how policymakers and young adult drinkers construct binge drinking narratives. An analysis of the lexicogrammar of a text (i.e., the words and phrases that are used and how those words and phrases are positioned within the clause) can provide insights into how the Speaker/Writer construes events and perceives the world, while also shedding light on the ways in which ideas, categories, identities and beliefs are constructed, reinforced and reconfigured through language. Adopting such an approach allows us to compare young adult drinkers' binge drinking narratives with equivalent narratives crafted by policymakers tasked with reducing alcohol harms. In this paper, we used linguistic analysis to highlight similarities and differences in how young adult drinkers and English policymakers construct binge drinking narratives. We had three research questions:

RQ1: Which semantic domains are used by both policymakers and young adult drinkers to construct alcohol consumption narratives?

RQ2: Which semantic domains are only used by policymakers to construct alcohol consumption narratives?

RQ3: Which semantic domains are only used by young adult drinkers to construct alcohol consumption narratives?

## 2 METHODS

### 2.1 Design

We conducted a secondary linguistic analysis to compare the narratives presented in two corpora: (i) the three most recently published English alcohol‐specific policy documents (Alcohol Harm Strategy [6]; Safe, Sensible, Social [7]; The Government's Alcohol Policy [8]), collectively labelled The Policy Corpus (TPC); and (ii) drinkers' accounts of their own alcohol consumption [13] labelled The Drinker Corpus (TDC); TPC contains 78,254 words, TDC contains 39,912 words.

### 2.2 Linguistic analysis

The text analysis software Wmatrix [27] and Sketch Engine [28] were used to explore the two corpora. Wmatrix was used to identify key semantic domains; the software automatically assigns each word in TPC and TDC a semantic tag according to the UCREL Semantic Analysis System (USAS).^2^ The USAS semantic tag set contains 21 general semantic domains (indicated by a letter) and a further 211 related sub‐domains (indicated by a number and sometimes a plus or minus symbol). For example, the semantic domain ‘Food and farming’ is assigned the letter F. This domain has four sub‐domains, represented by numbers, namely: ‘Food’ (F1), ‘Drinks and alcohol’ (F2), ‘Smoking and non‐medical drugs’ (F3) and ‘Farming and horticulture’ (F4). The sub‐domain ‘Drinks and alcohol’ (F2) has two further sub‐categories (indicated by the plus or minus symbol) to reflect polarity: F2+ represents ‘Excessive drinking’ while F2− represents ‘Not drinking’.

Wmatrix can identify semantic domains that are statistically overused in corpora by comparing them against a much larger reference corpus, for example, the British National Corpus (BNC)—a 100‐million‐word corpus containing a range of written and spoken texts dating from 1969 to 1994.^3^ The log‐likelihood (LL) score is used to calculate the statistical significance (or ‘keyness’) of a semantic domain. A LL score of 6.63 (which corresponds to a *p*‐value of 0.05, [29]) or above is considered significant. This type of analysis reinforces our intuitions regarding the ‘aboutness’ [30] of a text, that is, the various topics and themes within the discourse. However, it also alerts us to topics and themes in the discourse that were not completely obvious through a qualitative reading of the data (see e.g., [21]). A key‐semantic‐domain analysis, therefore, provides an overall sense of the content of our data while also pointing to possible topics (or domains) that may be worth further investigation.

Sketch Engine is used to examine some of the words that belong to semantic domains in more detail to see what the language might reveal. Sketch Engine contains two key functions we used to understand language in context: (i) the concordance function used to carry out a qualitative analysis of the words in context; (ii) the collocation function to identify words that are statistically likely to occur immediately to the left or right of key words to discern the wider discourse context.^4^ To measure statistical significance, the LogDice score is used [32]—the higher the score, the stronger the relationship between the two words.

Corpus linguistics, as a methodology for exploring how language is used by individuals and groups in different social situations, takes language out of context and reorganises it (based on quantitative investigations) to identify patterns—frequently occurring words, phrases or grammatical structures—that would be very difficult to notice through a traditional reading of the texts. By constantly moving between a micro‐ and macro‐level analysis of the language, it is possible to notice both what is typical and unusual about a text or texts. Sometimes the results may simply verify our intuitions. More often than not, however, a corpus analysis will reveal new insights into how language is used in a particular social context. While there is an element of us choosing to focus in on particular semantic domains, following our assumptions that these are the most important domains to explore in alcohol narratives, these choices are made following a data‐driven process, that is, they are based on the semantic domains that are statistically significant within and across both corpora.

In this analysis we adopted a Systemic Functional Linguistics (SFL) approach to help make sense of some of the quantitative (corpus) findings. SFL provides a structured framework through which to analyse, interpret and discuss language. This approach views language as a network of systems; meaning is achieved through the selections (or linguistic choices) that are made at each point in the network. In other words, SFL is concerned with choices language users make, consciously or subconsciously, and how these construe meanings in situational and cultural contexts. These semiotic processes are best understood in terms of three metafunctions—the ideational, interpersonal and textual. Here, we focus on ideational meaning, which relates to the way in which language is used to ‘talk about the world’. This could be ‘the external world – things, events, qualities etc. – or our internal world – thoughts, beliefs, feelings etc.’ [33, p. 86]. Specifically, we examined the way in which the lexicogrammar of the clause organises experience into participants (the things or people that are involved in the event), and processes (the verbal group which tells us about the event) [34, p. 86–88]. In SFL this is described as the system of transitivity. In this system there are different process types for construing different domains of experience. Material processes, for instance, are those which relate to outer experience (‘what we experience as going on ‘out there’, in the world around us’) [34, p. 170] and usually describe an event or action, for example work, give and go. In contrast, mental processes are those which relate to inner experience (‘what we experience as going on inside ourselves, in the world of consciousness’) [34, p. 170] and usually describe emotions, thoughts or perceptions, for example, see, know and think. There are six categories of process type: material, mental, behavioural, verbal, relational and existential.

In this paper, we examine the language of our two corpora through the lens of the ideational metafunction. In so doing, we recognise that we are restricting our analysis to noun and verb groups. Nevertheless, for the purpose of this study, the ideational metafunction allowed us to explore how things (people, groups, ideas, etc.) are named and described and how events and experiences are construed. Specifically, the ideational metafunction provides a linguistic framework through which to examine how policymakers and young people conceptualise and experience alcohol use as evidenced through their use of language.

### 2.3 Source material

In this paper, we make comparisons between two datasets (TPC; TDC). Our intention, in comparing TPC and TDC datasets, is to consider how they are similar and different from one another. In their own ways, they are constructing narratives about the same phenomenon, binge drinking. Further details about the corpora are provided next.

#### 2.3.1 The policy corpus

Justification for combining the documents includes: (i) in all three documents the 18‐ to 24‐year‐old group (young adults) is identified as being both vulnerable (they are most at risk of the harms of alcohol misuse) and problematic (they are the main contributors to the anti‐social drinking culture). Messaging campaigns such as Know your Limits [9] encourage young people to reflect on their behaviour when drinking; (ii) the key messages to the target group largely remained the same in all three documents: stay within recommended limits for units, binge‐drinking is harmful, be sensible, or responsible. To provide further justification for combining the policy documents we used Wmatrix to identify the top 20 key semantic domains in each document (see Table S1, Supporting Information). Domain F2, ‘Drinks and alcohol’ is in first place position for all three strategies with LL scores of <3603.71>, <5160.31> and <1738.71>. The significant ‘overuse’ of words relating to drinks and alcohol is to be expected given the nature of the texts being analysed. The domain G2.1 ‘Crime’, also scores consistently high across the three documents (3rd position in the 2004 and 2007 strategies and 4th position in the 2012 strategy) and more prominent than B2 ‘Health and Disease’, which gains prominence over time (moving from 12th in 2004, to 6th in 2007 and 2nd in 2012).

#### 2.3.2 The drinker corpus

To capture young adult drinkers' alcohol narratives, we used data previously reported in Burgess et al. [13]. This dataset was collected from a sample of 512 young adults (mean age = 23.59 (6.43), 351 female, 153 male, 6 prefer not to say, 2 blank) in November 2015. They were asked to write, in their own words, how they experienced approaching and exceeding the threshold of drinking too much alcohol, which can be likened to binge drinking. Participants were asked to answer if they had an intuitive sense of what would constitute too much alcohol (either in terms of the way in which drink makes you feel or in terms of an absolute amount of alcohol)? If they answered yes, they were asked four further questions: (i) to describe how you established your own personal intuitive sense of too much and if this had changed over time; (ii) whether it (sense of too much) was something that remains constant across different situations or does it fluctuate according to situation?; (iii) Imagine you are actually drinking and that you approach, but do not exceed, your own personal intuitive sense of ‘too much’. Can you describe the feelings thoughts, and just generally what it is like to approach, but not exceed, your own personal sense of too much?; (iv) Imagine you are actually drinking and that you exceed your own personal intuitive sense of ‘too much’. Can you describe the feelings thoughts, and just generally what it is like to exceed, your own personal sense of too much? We used a theoretical framework for understanding first‐person experiential states based on Gallagher and Zahavi's conceptualisation of embodiment as a principle of experience, existing on a positive–negative continuum of physical and affective states [35, 36]. Participants' open‐ended descriptions of approaching and exceeding a personal threshold of too much alcohol were thus analysed to determine the nature of the experience in relation to: positive or negative embodiment, by ongoing awareness of the effect of alcohol, by the ability to exert control, and by positive or negative views of self and social interactions.

Overall, we conducted a linguistic analysis of TPC and TDC to explore which semantic domains were used by both policymakers and drinkers, to identify domains and words important only to policymakers, and domains and words important only to drinkers.

## 3 RESULTS

### 3.1 Key semantic domains found in both corpora

Five of the top 20 semantic domains were found to exist in both corpora (see Table 1). F2 ‘Drinks & alcohol’ was the top domain in both corpora with an LL score of <8818.92> in TPC and <4327.45> in TDC. Other prominent domains are: F2++ ‘Excessive drinking’, B2 ‘Disease’, A2 ‘Cause & effect/connection’ and X2.2+ ‘Knowledgeable’. Having identified key domains in both TPC and TDC, we next examined words that belonged to these domains to see how policymakers and young adult drinkers talk about these subjects.

**TABLE 1:** Comparison of semantic domains in The Drinker Corpus and The Policy Corpus.

The Policy Corpus			The Drinker Corpus		
Tag	Semantic domain	LL	Tag	Semantic domain	LL
**F2**	**Drinks and alcohol**	**8818.92**	**F2**	**Drinks and alcohol**	**4372.45**
G2.1‐	Crime	1220.54	N5.2+	Exceed; waste	1938.83
A1.1.2	Damaging and destroying	1049.83	**F2++**	**Excessive drinking**	**1296.73**
X7+	Wanted	1005.67	**B2‐**	**Disease**	**1156.8**
S8+	Helping	920.96	X2.1	Thought, belief	1080.31
G1.1	Government	806.6	A2.1+	Change	908.79
B2	Health & disease	793.5	N5.1	Entirety; maximum	631.47
B3	Medicines and medical treatment	760.17	A6.2+	Comparing; usual	576.27
**A2.2**	**Cause and effect/connection**	**672.72**	E4.1+	Happy	438.55
G2.1	Law and order	629.66	**A2.2**	**Cause and effect/connection**	**384.95**
S2	People	603.19	A13.6	Degree: diminishers	356.83
**F2++**	**Excessive drinking**	**574.3**	T2+	Time: beginning	356.48
**X2.2+**	**Knowledgeable**	**543.77**	T2‐	Time: ending	331.37
I2.2	Business: selling	498.33	A13.3	Degree: boosters	326.27
**B2‐**	**Disease**	**464.24**	*E1*	*Emotional actions, states and processes*	*231.26*
G2.2‐	Unethical	424.97	**X2.2+**	**Knowledgeable**	**226.68**
X2.4	Investigate, examine, test, search	422.89	E4.1‐	Sad	215.63
T1.1.3	Time: future	410.63	A13	Degree	198.63
*S1.1.1*	*Social actions, states and processes*	*408.17*	X2	Mental actions and processes	197.38
I4	Industry	393.34	A1.7‐	No constraint	164.03

*Note*: Similar domains highlighted in bold. The Italics indicate semantic domains that appear in one corpus only.

Abbreviation: LL, log‐likelihood.

Table S2, shows words that were tagged as belonging to the shared domains. Focusing on F2++ ‘Excessive drinking’, the most common word in both corpora is ‘drunk’, with frequencies of <71> and <179> in TPC and TDC, respectively; ‘drunkenness’, ‘drunken’ and ‘intoxicated’ also occur in both corpora. However, while the word ‘binge’ has a frequency of <37> in TPC, it is not used in TDC—the word ‘tipsy’ is more common, with a frequency of <30>.

B2—‘Disease’ is a key topic for both policymakers and young adult drinkers. In TPC, this domain contains words relating to long‐term conditions and mental illness; words such as ‘disorder’ <180>, ‘liver disease’ <18>, ‘ill health’ <14> and ‘mental illness’ <11>. In TDC, the domain contains words relating to short‐term physical symptoms; words such as ‘sick’ <169>, ‘dizzy’ <56>, ‘hangover’ <21> and ‘light‐headed’ <18>. Overall, policymakers view alcohol in terms of long‐term consequences, while drinkers are concerned with short‐term consequences of drinking. To explore this idea further, we took a closer look at the domain A2.2 ‘Cause & effect / connection’, which was also key in both corpora, with LL scores of <672.72> in TPC and <384.95> in TDC. We focus on the word ‘consequences’ as this had the same relative frequency in both corpora.

#### 3.1.1 A closer look at ‘consequences’

There are 36 instances of ‘consequences’ in TPC and 20 in TDC. In the TPC, concordance lines showed that all 36 occurrences refer to negative consequences. Partly, this is to be expected as consequence tends to have negative semantic prosody [37], meaning it attracts words with negative associations. Most often, the consequences themselves are not explicitly stated (<33> occurrences). Instead premodifiers such as ‘adverse’ <3>, ‘serious’ <2>, ‘harmful’ <2>, ‘criminal’ <2> and ‘severe’ <1> are used (examples 1–4 in Table 2). In the three instances where the consequences are stated, they are extreme: ‘domestic violence’, ‘assault or neglect of children’, ‘injuries’ and problems at school. In addition, almost a third (11 out of 36) of all occurrences of ‘consequences’ in TPC are in Object position. The verbs in these clauses are often to do with managing consequences (verbs such as ‘deal with’ <5>, ‘manage’ <4> and ‘tackle’ <2>, examples 5–7 Table 2). Other verb phrases relate more to the messaging and communication of consequences: ‘raise/increase awareness of’ <2>, ‘emphasise’ <2>, ‘provide information on’ <1> and ‘warn about’ <1> (examples 8–10 Table 2). There are only two instances where a human participant is mentioned in relation to ‘consequences’: ‘supporting those [people] who suffer adverse consequences’ and ‘some [people] will suffer very serious consequences’.

**TABLE 2:** Example concordance lines.

Ref	Example	Source
1	But some will suffer very **serious consequences**	2004 strategy
2	a colleague who fails to turn up for work, through to much more **severe consequences**	2007 strategy
3	Too many people drink in this way without realising the **harmful consequences**	2007 strategy
4	and aims to raise awareness among young drinkers of the **adverse consequences**	2004 strategy
5	Much of the strategy we have outlined hinges on raising awareness of alcohol and **dealing with** its **consequences** within existing activity	2004 strategy
6	minimising the harm caused by alcohol misuse and working with local agencies to help **tackle** the **consequences**	2004 strategy
7	A financial contribution from the industry towards **managing** the crime and disorder **consequences** of alcohol misuse	2004 strategy
8	The campaign played on the vulnerability of binge drinkers and **emphasised** both the physical and criminal **consequences** that can arise from irresponsible alcohol consumption.	2007 strategy
9	in general the industry **provides** little information on the possible **consequences** of alcohol misuse	2004 strategy
10	Giving accurate information about the products it sells and **warning** about the **consequences**	2004 strategy
11	Absolutely wild, **every thing is fun**, nothing I do has **consequences** or matters, it's truly wonderful everyone is very funny, I can dance **i feel in control** of myself for doing this	23 year old female
12	more **adventurous, more outgoing and ‘brave’** (stupid), less likely to think of **consequences**, more **euphoric Feeling a little out of control**, possibly feeling nauseous, being aware that I might not do things	20 year old male
13	I don't know my limits so much and **continue to drink to feel as numb as possible** without thinking of the negative **consequences**	20 year old female
14	It is situation dependent, was developed from past experience of the **consequences** (**vomiting, prolonged hangover** etc) It has changed over time	23 year old male
15	I perhaps wouldn't be too bothered at the time because i would be drunk but the **consequences** would be present the next day such as **feeling very ill, feeling embarrassed** and i would also probably waste an entire day	18 year old male
16	maybe considering **consequences** a bit less, so **i will think less before i do or say something** compared to sober state	27 year old female
17	**Feel happy, relaxed and more carefree**. It's a nice feeling, like you feel happy and giggly	20 year old female
18	**I** chat more, get a little louder, start not to care so much, **feel more liberated, up for having fun I feel confidence, giggly, loud and more talkative**	21 year old female
19	I don't like to lose control when I'm drinking so if **I** start to **feel sick** or feel I'm getting drunk quite quickly then I tend to slow down	21 year old female
20	I might worry a little about if **I'd feel ill** tomorrow or if it would make me behave differently	30 year old female
21	However in the morning **I'd feel guilty and upset** with myself and worried about my health and control	19 year old female
22	**I** would **feel embarrassed and helpless**	19 year old female
23	**I feel a bit guilty but I'm having a good time I'm not too worried**	18 year old female
24	My teeth feel funny, my face gets hot, and **I feel fuzzy** I start to question whether I should go home	39 year old male
25	Too much is when **I start feeling tipsy**	23 year old female

*Note*: The bold formatting is to highlight the key parts of the text that is referred to in the main body of the text.

In contrast, with the TDC there are zero instances of these premodifiers found in TPC; instead drinkers talk about ‘possible’, ‘potential’, ‘physical’ and ‘immediate’ consequences (<4> occurrences). In more than half (11/19) of all instances drinkers refer to ‘not caring’ or ‘not thinking’ about consequences. In <5> instances, the interviewees talk about having a sense of freedom and euphoria or feeling adventurous and brave (examples 11–12, Table 2). This appears to be a significant part of the enjoyment of binge drinking—that is, alcohol causes them to not worry about any consequences. Typically, not caring or thinking about consequences is discussed alongside very powerful and positive experiences of binge drinking. Conversely, in <6> instances, the consequences of binge drinking, on the surface at least, appear to be unpleasant (feeling sick, spinning head, problems with vision etc.). However, these too are framed positively (i.e., the participant knows the negative consequences, but they do not care). In these occurrences, not caring about consequences is part of the appeal of drinking alcohol (example 13, Table 2). In instances where the consequences are clearly perceived as being negative, drinkers refer to short‐term physical symptoms such as vomiting, struggling to speak, feeling ill or feeling confused (five occurrences), embarrassment (three occurrences) and acting in a way that is not typical (one occurrence)—(examples 14–16, Table 2).

Overall, ‘consequences’ in TPC tend to be longer‐term, vague or extreme. On the rare occasions when consequences are explicitly stated they tend to be serious things to do with crime and violence. Consequences are to be tackled and managed and the public needs to be warned or educated about them. Consequences are to be suffered. In TDC, consequences are immediate, short‐term and mostly have positive associations.

### 3.2 A semantic domain used only by policymakers

Table S3, shows words that are associated with the domain S1.1.1 ‘Social actions, states & processes’, which is prominent in TPC, but is not in the top 20 domains in TDC. The most frequent word is ‘behaviour’ which has a relative frequency per hundred words of <0.28>. Table S4, shows collocates for ‘behaviour’ in TPC and TDC. The strongest collocate in TDC is the possessive pronoun ‘my’ (with a LogDice score of <9.85>), while in TPC it is the pre‐modifying adjective ‘anti‐social’/‘antisocial’/‘Anti‐Social’ (with LogDice scores of <12.12>, <11.6> and <10.89>, respectively). Also, of the 19 collocates for ‘behaviour’ in TPC, many are pre‐modifying adjectives with negative connotations: ‘disorderly’, ‘irresponsible’, ‘rowdy’, ‘drunken’, ‘offending’, ‘unacceptable’ and ‘criminal’.

As these observations are perhaps unsurprising, we used Sketch Engine's concordance function to examine the word in its wider discourse context^5^, and better understand the meaning and use of the term ‘behaviour’ in the two datasets. We used a SFL approach (e.g., [34]) to analyse a sample of concordance lines for ‘behaviour’ in both datasets (50 randomly selected from TPC and all 29 occurrences from TDC). Results are presented in Table 3.

**TABLE 3:** Clause analysis of ‘behaviour’ in the policy corpus.

Participant role	Process type	Participant role
we (10) government (1) the Home Office (1)	Material processes: TACKLE (4) CHANGE (2) COMBAT (1) CHALLENGE (1) DEAL WITH (1) ENCOURAGE (1) REDUCE (1) Mental processes: WANT (1)	anti‐social behaviour (6) behaviour (3) unacceptable behaviour (2) acceptable behaviour (1)
alcohol education programmes (2) education interventions (1) one‐to‐one work (1)	Material processes: ADDRESS (2) PROMOTE (1) Relational processes: BE (1)	behaviour (1) behaviour change (1) changing behaviour (1) offending behaviour (1)
Acceptable Behaviour Contracts (1) Anti‐social Behaviour Orders (1) Fixed Penalty Notices (1) Youth Justice Board (1)	Material processes: CLAMP DOWN ON (1) ENGAGE (1) PROTECT (1) REDUCE (1)	anti‐social behaviour (2) behaviour (1) disorderly behaviour (1)

Of the 50 occurrences of ‘behaviour’ randomly selected from TPC, 10 are noun phrases used as (sub‐) headings or in bullet‐point lists (e.g., ‘Crime and anti‐social behaviour harms’), three are part of a noun phrase in Subject position within the clause (e.g., ‘Attitudes and behaviour are inextricably linked to the surrounding culture’) and the remaining 37 occurrences are part of a noun phrase in Object position (e.g., ‘We will work in partnership with the industry to reduce anti‐social behaviours’). Of these 37 occurrences, three main types of participant are typically found in Subject position: the government (‘we’, ‘government’, ‘the Home Office’); educators (‘alcohol education programmes’, ‘education interventions’, ‘one‐to‐one work’) and legislative organisations or processes (‘Acceptable Behaviour Contracts’, ‘Anti‐social Behaviour Orders’, ‘Fixed Penalty Notices’, ‘Youth Justice Board’). These clauses mostly contain what is described in SFL terms as a material process. Material process clauses construe outer experience—physical actions such as making, doing, getting and so on. In the examples being discussed here, the most common material process is ‘tackle’, followed by ‘change’ and ‘address’.^6^

In summary, ‘behaviour’ in TPC is mostly pre‐modified by an adjective expressing negative sentiment: ‘anti‐social’, ‘disorderly’, ‘unacceptable’. It is typically part of a material process clause in which the main Actor is the government, educators or legislation of some description, whose aim is to ‘tackle’, ‘change’ ‘address’ and ‘clamp down on’ ‘anti‐social’ and ‘disorderly’ drinking behaviours of individuals. Individual behaviour always needs to change.

### 3.3 A semantic domain used only by drinkers

The domain E1 ‘Emotional actions, states & processes’ is prominent in TDC but is not in the top 20 domains in TPC. Table S5 lists words associated with this domain. The most common word is ‘feel’ with a relative frequency of <0.08>. The verb ‘feel’—a mental process—is not very frequently used in TPC (only <11> occurrences/<119.51>p/million words) and is mostly used when discussing perceptions of alcohol among the public (e.g., ‘Most people (78%) feel informed about the risks of alcohol’). In TDC, feel is frequently used (<783>occurrences, 17,569.84 p/million words) in the context of discussing the effects of alcohol consumption. Table 4 lists collocates immediately to the right of ‘feel’ in TDC.

**TABLE 4:** Collocates for FEEL in the drinker corpus (1R).

Collocate	Raw freq.	LogDice
*sick*	65	11.1
like	60	11.05
very	27	9.92
a	47	9.89
*ill*	21	9.71
out	24	9.66
**happy**	19	9.52
as	20	9.27
that	24	9.27
**good**	16	9.23
quite	15	9.2
*dizzy*	15	9.19
*drunk*	16	9.01
*guilty*	12	8.94
tipsy	12	8.91
slightly	12	8.9
more	15	8.81
the	22	8.79
*bad*	10	8.64
in	13	8.51
really	9	8.43
too	12	8.25
**relaxed**	7	8.15
**light**	7	8.14
*tired*	7	8.12
*nauseous*	6	7.95
**safe**	6	7.93
**better**	6	7.9
when	8	7.89
proud	5	7.7
*unwell*	5	7.68
**confident**	5	7.66
it	7	7.63
I	21	7.58
less	5	7.55
**myself**	5	7.42
*embarrassed*	4	7.36
*awful*	3	6.96
*confused*	3	6.96
my	5	6.95
completely	3	6.95
fuzzy	3	6.95
any	3	6.87
much	4	6.55
and	5	6.37

*Note*: 1R = words that appear one word to the right of the search word, for example, feel *happy*. The bold text indicates positive terms. The Italics denote negative terms.

What is perhaps unexpected when looking at the list of collocates are the number of words (mostly adjectives) that appear to describe positive feelings and experiences: ‘happy’, ‘good’, ‘relaxed’, ‘light’, ‘safe’, ‘better’, ‘confident’ and ‘myself’. Indeed, there are almost as many ‘positive’ words as there are ‘negative’ ones.^7^ A closer look at the language in context reveals that while the ‘positive’ words tend to describe emotional states (such as feeling ‘proud’, ‘confident’ or ‘relaxed’—examples [17, 18], Table 2), the ‘negative’ ones mainly describe physiological states (such as feeling ‘ill’, ‘dizzy’, ‘tired’ and ‘nauseous’—examples [19, 20], Table 2). Only three of the ‘negative’ words refer to emotional states, namely the adjectives ‘guilty’, ‘embarrassed’ and ‘confused’—examples [21, 22, 23], Table 2. A further two adjectives in Table 4, ‘tipsy’ and ‘fuzzy’, could be construed as either positive or negative, however, a closer look at these words in context shows that ‘feeling fuzzy’ or ‘feeling tipsy’ are both used as indicators to stop drinking—examples [24, 25], Table 2.

In sum, young adult drinkers report significant positives to binge drinking, centred around positive feelings and enhanced emotional and psychological states. Negative feelings associated with binge drinking are mainly centred around physiological changes (being sick, ill, nauseous etc.). The negative emotional impact of drinking seems to centre around feelings of guilt and embarrassment.

## 4 DISCUSSION

This paper reports the results of a linguistic analysis that compares the lexicogrammar of the last three English alcohol policy documents (TPC) with open‐ended survey responses from a sample of English young adult drinkers (TDC). We found these corpora share similarities in some of the superordinate categories they reference, for example, both frequently mention drinking and diseases linked to alcohol. However, examining the words used within these categories, there are differences between the two corpora. In addition, there were also differences in superordinate categories between the corpora.

Linguistic analyses identified differences in words used by policymakers and young adult drinkers to describe binge drinking. While both groups used the word ‘drunk’, ‘binge drinking’ was used by policymakers but not young adults, who instead used the term ‘tipsy’. Drinkers likely view ‘tipsy’ as a favourable state of intoxication; ‘tipsy’ suggests someone who has not exceeded their subjectively defined ‘tipping point’—the point in which they feel out of control [38, 39]. In contrast, binge drinking has negative connotations including feeling out of control [40, 41]. Gough et al. [42] argue that to maintain a morally acceptable identity in the face of conflicting information people often make efforts to present themselves as good citizens, which is similar to Melia et al.'s [43] idea about drinking falling on the right side of the line between good and bad. Such discursive strategies drive stigma as a key issue in alcohol language and behaviour; heavy drinkers may draw on more severe ‘alcoholism’ stereotypes to maintain their own non‐problematic drinking identity [44].

Another difference between the corpora is in the timeframe associated with alcohol harms. The TPC corpus primarily talks about long‐term harms, liver disease, disorders, whereas the TDC talks about short‐term harms, hangovers, nausea. While it is understandable that policies focus on long‐term harms, research shows that young adults are more attuned to social losses than long‐term health consequences [45]. When writing policies to reduce young adults' binge drinking it is advisable to focus on short‐term harms in messages presented to young adults.

Our analysis also allowed us to highlight narratives frequently used in one corpus but not the other. In the TPC, behaviour is always bad, and should be reduced, or clamped down on. Behaviour should be modified with the support of organisations, industry, educators. We found evidence of material processes being used in policies, which is a strength of using linguistic analysis. In the TDC, ‘feel’ was used to describe positive and negative feelings about alcohol. According to Cox and Klinger's [16] incentive motivation model, two of the four main motives for drinking alcohol are to: (i) enhance the way you feel; or (ii) cope with negative emotions (feelings). Enhancement and coping motives are consistent correlates of young adults' binge drinking [21]. Beyond this recognition of the importance of feelings, we noted that young adult drinkers reported positive psychological effects of binge drinking and negative physiological effects. Therefore, using these negative effects as part of prevention strategies within policies is challenging because psychological effects are typically experienced before physiological effects.

### 4.1 Implications and future research

All the policy documents we analysed talked about the need for improved messaging to young adult drinkers. We have previously discussed the disconnect between messaging and drinkers [11] and the need for policymakers and researchers to collaborate more [46]. Our current analysis highlights this issue; in the ‘Social actions, states and practices’ domain that appeared in the TPC corpus, there was no mention of working with the target group, that is, young adult binge drinkers. Instead, policies talked about the government working with educators and health organisations. We think this is a key issue to address when writing future policies as current approaches, that is, telling people to ‘know your limits’ or ‘drink responsibly’ are not effective at reducing binge drinking among young adult drinkers. Researchers, policymakers, stakeholders, and drinkers, should all be part of the conversation to bring about meaningful reductions in binge drinking at the population‐level [24].

Policymakers are encouraged to acknowledge the reality that feelings (i.e., emotional states) can drive alcohol use. That is ‘binge drinking’ is a behaviour people sometimes do because they want to feel something, to be part of a ‘big night out’ or forget about their worries. Researchers have raised the issue of recognising that drinking is done because it is fun [47] and the need to factor this in when designing health campaigns [48]. Oldham et al. [10] asked increasing and higher‐risk UK drinkers about interventions to reduce consumption and found that interventions that interfered with positive feelings were judged as less appealing. Alcohol health risk messages are also most likely to be rejected by those with the highest level of consumption [12], and that those with the highest level of consumption often possess fewer resources, such as social support, to help them to reduce their consumption [49]. Such findings present further key questions for how behaviour change may be enacted via campaigns that require awareness of messages focused on changing individuals' behaviour.

We also believe there is a need to widen the focus of alcohol policy beyond young adult drinkers. Young adults in England [50], and globally [51], are drinking less than previous generations. In contrast, middle‐ and older‐aged adults are consuming alcohol more frequently and in higher amounts than previously. While mid‐life and older adults drinking behaviours have received less attention than young adult drinking, researchers are attempting to redress the balance in an emerging body of literature [49, 52, 53, 54]. It is important that when writing future alcohol policies, policymakers should focus their attention on these older groups as their drinking patterns have the potential to lead to expensive treatment for long‐term health conditions (liver disease, cancer, coronary heart disease, type 2 diabetes). While young adults' drinking is more visible than other groups, and this may be why they remain the focus of policymakers and researchers, it is unarguable that middle‐aged and older adults' drinking has a major impact on health services and deserves more attention. Existing messages around cancer and other long‐term diseases may resonate more with older drinkers [54] compared with young adult drinkers. Finally, we note that literature comparing short‐and long‐term framing of health messages [55, 56] is consistent with our finding that young adult drinkers have a focus on short‐term effects of drinking that is noticeably absent from the long‐term focus present in policy documents.

### 4.2 Limitations

A potential limitation of this paper is that it compares text from datasets with different aims: The TPC is communicating narratives to meet policy briefs, while the TDC is how drinkers responded to items created to stimulate discussion. Nevertheless, comparing these two databases makes it possible to highlight important differences not articulated in past research. Our analysis has identified areas that might be worth considering in more detail in order to produce policy better suited to changing binge drinking. A further limitation is that the policies we included are outdated and refer to past government priorities. However, until such time as the English government publishes new alcohol‐specific policy, these are the three most recent English policies. We believe that using multiple policies allows for a more comprehensive overview of policy than if we were to use one policy. We hope that by showing the potential of linguistic analysis on different corpora we can encourage their use by other researchers.

## 5 CONCLUSIONS

Young adult drinkers and policymakers use different languages of alcohol. Drinkers focus mainly on short‐term harms and talk about embodied feelings. Policymakers focus on health conditions and tackling ‘bad’ behaviour. It is time for researchers to work with policymakers and drinkers to develop policies and associated messages that are more likely to reduce alcohol consumption.

## AUTHOR CONTRIBUTIONS

Emma Moreton conceived the idea for the study, ran all analyses, co‐wrote the methods and results sections of the paper, and provided feedback on the introduction and discussion sections of the paper. Emma Davies collected data and conceived questions for a dataset used in the study, co‐wrote the methods section of the paper and provided feedback on the paper. James Morris co‐wrote the introduction and provided feedback on the paper. Richard Cooke conceived the idea for the study, co‐wrote the introduction and results sections of the paper, wrote the discussion section of the paper, and provided feedback on the method sections of the paper.

## CONFLICT OF INTEREST STATEMENT

The authors declare no conflicts of interest.

## Supporting information


**Table S1.** Key semantic domains—similarities across the three alcohol strategies.
**Table S2.** Words tagged as belonging to the five key domains that are in both The Policy Corpus and The Drinker Corpus.
**Table S3.** Collocates for Behaviour in The Policy Corpus and The Drinker Corpus (1L).
**Table S4.** Words in The Policy Corpus belonging to S1.1.1 social actions, states and processes.
**Table S5.** Words in The Drinker Corpus belonging to E1 emotional actions, states and processes.

## Data Availability

Research data are not shared.

## Endnotes

^1^
1 unit = 8 g of pure alcohol.

^2^
UCREL Semantic Analysis System (USAS) (https://ucrel.lancs.ac.uk/usas/).

^3^
As TPC contains written texts and TDC contains spoken texts, we use the BNC Written Sampler sub‐corpus to calculate key semantic domains in TPC and we use the BNC Spoken Sampler sub‐corpus for TDC.

^4^
Collocation ‘is a psychological association between words…and is evidenced by their occurrence together in corpora more often than is explicable in terms of random distribution’ [31, p. 5].

^5^
Collocation ‘is a psychological association between words … and is evidenced by their occurrence together in corpora more often than is explicable in terms of random distribution’ [31, p. 5].

^6^
Note that ‘address’ can be categorised in different ways (as a verbal process, for e.g.). Here it is being used in the sense of ‘do something about something’, so it has been categorised as material.

^7^
Note, the adjective ‘proud’ has not been categorised as a ‘positive’ word as this is typically used in the context of NOT drinking (e.g. ‘I feel proud of myself for not drinking more than I should’).
