# Supplementary material for: The language of alcohol: Similarities and differences in how drinkers and policymakers frame alcohol consumption
Source: Drug Alcohol Rev. 2025 Apr 10;44(4):1194–206. doi: 10.1111/dar.14056 (PMC12117306; doi:10.1111/dar.14056)
Supplement: Supplementary file 1 — Table S1. Key semantic domains—similarities across the three alcohol strategies. Table S2. Words tagged as belonging to the five key domains that are in both The Policy Corpus and The Drinker Corpus. Table S3. Collocates for Behaviour in The Policy Corpus and The Drinker Corpus (1L). Table S4. Words in The Policy Corpus belonging to S1.1.1 social actions, states and processes. Table S5. Words in The Drinker Corpus belonging to E1 emotional actions, states and processes. [file DAR-44-1194-s001.docx]

Table S1. Key semantic domains – similarities across the three alcohol strategies

| **Tag** | **Semantic domain** | **2004 LL** | **Rank** | **2007 LL** | **Rank** | **Change in rank** | **2012 LL** | **Rank** | **Change in rank** |
| --- | --- | --- | --- | --- | --- | --- | --- | --- | --- |
| F2 | Drinks and alcohol | 3603.71 | 1^st^ | 5160.31 | 1^st^ | = | 1738.71 | 1^st^ | = |
| G2.1- | Crime | 624 | 3^rd^ | 572.62 | 3^rd^ | = | 174.39 | 4^th^ | -1 |
| B3 | Medicines & medical treatment | 403.47 | 5^th^ | 285.85 | 12^th^ | -7 | 138.15 | 8^th^ | +4 |
| A2.2 | Cause & effect / connection | 391.9 | 6^th^ | 224.07 | 15^th^ | -9 | 111.22 | 10^th^ | +5 |
| G2.1 | Law & order | 370.57 | 8^th^ | 217.41 | 17^th^ | -9 | 95.55 | 14^th^ | +3 |
| S8+ | Helping | 367.42 | 9^th^ | 439.57 | 4^th^ | +5 | 184.9 | 3^rd^ | +1 |
| G1.1 | Government | 349.3 | 10^th^ | 352.89 | 10^th^ | = | 166.36 | 6^th^ | +4 |
| B2 | Health & disease | 259.14 | 12^th^ | 413.82 | 6^th^ | +6 | 278.79 | 2^nd^ | +4 |
| F2++ | Excessive drinking | 222.53 | 15^th^ | 360.7 | 8^th^ | +7 | 106.76 | 11^th^ | -3 |
| B2- | Disease | 179.95 | 17^th^ | 267.82 | 13^th^ | +4 | 57.23 | 16^th^ | -3 |
| I2.2 | Business: selling | 173.63 | 19^th^ | 204.5 | 18^th^ | +1 | 172.42 | 5^th^ | +13 |
| S2 | People | 146.73 | 20^th^ | 378.42 | 7^th^ | +13 | 145.86 | 7^th^ | = |

LL, log-likelihood.

Table S2. Words tagged as belonging to the five key domains that are in both The Policy Corpus and The Drinker Corpus

| **Tag** | **Semantic domain** | **The Policy Corpus results** | **The Drinker Corpus results** |
| --- | --- | --- | --- |
| F2 | Drinks & alcohol | alcohol (1280), drinking (536), drink (173), drinkers (150), drinks (49), alcoholic (44), pubs (30), bar (19), drank (16), beer (14), drunk (13), bars (12), wine (11), pub (11), off-licence (9), drinker (8), off-licences (6), cider (5), beverage (5), beers (3), alcohol element (3), soft drinks (3), alcoholism (2), lagers (2), ciders (2), alcopops (2), on-licence (2), alcohol marketing (2), alcohol company (2), sips (2), | drink (342), drinking (263), alcohol (155), drinks (82), drunk (35), drank (20), sober (20), pub (19), beer (11), wine (10), sober up (10), bottle of wine (9), bar (8), glass of wine (7), drinking water (7), soft drink (7), glasses of wine (6), cider (6), soft drinks (6), beers (4), vodka (4), alcoholic (4), gin (4), ale (3), lager (3), sip (3), non alcoholic (3), booze (2), drinking based (2), alcohols (2), cocktails (2), drinker (2), bottles of wine (2), whisky (2), vs (2), |
| A2.2 | Cause & effect / connection | responsible (115), caused (66), related (55), impact (53), effects (43), cause (43), **consequences (36)**, result (34), linked (33), dependent (32), lead to (26), results (24), causing (22), based on (21), factors (20), causes (17), effect (16), produce (16), basis (16), due to (14), conditional (13), dependence (12), links (12), link (12), in relation to (12), factor (11), produced (10), impacts (9), influence (8), because of (8), why (7), reasons (7), depending on (6), implications (6), resulting (6), consequence (5), relate (5), determining (5), depends (4), relating (4), leads to (4), influences (4), in the light of (4), influencing (3), motivations (3), leading to (3), motivation (3), proactive (3), in light of (3), reason (3), determined (3), in response to (3), determine (3), producing (2), motivate (2), connection (2) linking (2), motivational (2), join (2), depend (2), depend (2), generates (2), joining (2), in respect of (2), interlinked (2), attributable (2), connections (2), derived (2), root cause (2), proportionate (2), affect (2) | depends (60), depending on (50), effects (20), **consequences (19)**, based on (18), effect (14), throw up (12), why (8), because of (7), depend (7), dependent (6), reason (6), due to (6), result (5), responsible (4), influenced (4), gets (3), factors (3), depending (3), caused (3), lead to (3), cause (3), consequence (2), causes (2), get (2), linked (2), throwing up (2), thrown up (2), affect (2), hence (2), |
| F2++ | Excessive drinking | drunk (71), binge (37), drunkenness (21), drunken (11), binge-drinking (8), intoxicated (2) | drunk (179), tipsy (30), drunkenness (7), intoxicated (5), drunken (3), drunker (2) |
| X2.2+ | Knowledgeable | Information (174), identification (65), identify (53), know (44), awareness (43), identified (37), identifying (22), informed (20), specialist (16), inform (15), aware (11), knowledge (10), warning (9), expertise (9), recognise (9), recognises (8), recognition (7), known (6), heard of (5), recall (5), recognising (4), experience (4), experts (4), identifies (3), remember (3), recognised (3), knowing (3), expert (3), informing (2), knowingly (2) | know (218), aware (71), remember (45), experience (30), experiences (25), knowing (21), conscious (13), recognise (8), awareness (8), warning (7), knowledge (4), remembering (3), remembered (3), knew (2), known (2) |
| B2- | Disease | disorder (180), patients (25), liver disease (18), injury (16), ill health (14), injuries (13), disease (11), mental illness (11), casualties (9), diseases (7), ill (6), stroke (6), heart disease (6), injured (6), health problems (5), illness (5), disorders (5), syndrome (4), coronary (4), cancer (3), mental health problems (3), health risks (3), mentally ill (2), symptoms (2), high blood pressure (2), diabetes (2), alcohol disorder (2), addiction (2) | sick (169), dizzy (56), ill (33), hangover (21), light headed (18), unwell (12), nauseous (9), dizziness (8), headache (8), giddy (7), vomit (5), hangovers (5), sickness (5), over the edge (4), vomiting (3), blackout (3), giddiness (3), blacked out (2), vomited (2), passed out (2), pass out (2), nausea (2), paranoia (2), paranoid (2), puke (2) |

Table S3. Collocates for BEHAVIOUR in The Policy Corpus and The Drinker Corpus (1L)

| **The Policy Corpus** | | | **The Drinker Corpus** | | |
| --- | --- | --- | --- | --- | --- |
| **Collocate** | **Raw freq.** | **LogDice** | **Collocate** | **Raw freq.** | **LogDice** |
| anti-social | 36 | 12.12 | my | 16 | 9.85 |
| antisocial | 24 | 11.6 | of | 3 | 6.91 |
| anti-Social | 14 | 10.89 |  |  |  |
| disorderly | 13 | 10.73 |  |  |  |
| irresponsible | 10 | 10.23 |  |  |  |
| rowdy | 8 | 10.11 |  |  |  |
| drinking | 22 | 9.96 |  |  |  |
| drunken | 6 | 9.68 |  |  |  |
| their | 11 | 9.54 |  |  |  |
| adults | 4 | 9.14 |  |  |  |
| change | 5 | 9.12 |  |  |  |
| offending | 4 | 9 |  |  |  |
| changing | 3 | 8.67 |  |  |  |
| unacceptable | 3 | 8.67 |  |  |  |
| social | 4 | 8.62 |  |  |  |
| criminal | 3 | 8.46 |  |  |  |
| on | 5 | 7.39 |  |  |  |
| and | 12 | 6.74 |  |  |  |
| the | 3 | 4.7 |  |  |  |

Note. 1L = words that appear immediately to the left of the word behaviour, e.g., *anti-social* behaviour.

Table S4. Words in The Policy Corpus belonging to S1.1.1 Social actions, states and processes

| **Word** | **Raw freq.** | **Rel freq.** |
| --- | --- | --- |
| behaviour | 214 | 0.28 |
| social | 141 | 0.18 |
| introduced | 19 | 0.02 |
| introduce | 15 | 0.02 |
| behaviours | 13 | 0.02 |
| introducing | 12 | 0.02 |
| in consultation with | 12 | 0.02 |
| socially | 10 | 0.01 |
| behavioural | 7 | 0.01 |
| treat | 4 | 0.01 |
| treating | 4 | 0.01 |
| lifestyle | 4 | 0.01 |
| behave | 4 | 0.01 |
| habits | 4 | 0.01 |
| lifestyles | 4 | 0.01 |
| manner | 4 | 0.01 |
| conduct | 3 | 0 |
| socialising | 3 | 0 |
| come into contact | 3 | 0 |
| contact | 3 | 0 |
| social services | 3 | 0 |
| engagement | 3 | 0 |
| proxy | 2 | 0 |
| traditionally | 2 | 0 |
| traditional | 2 | 0 |
| habits | 2 | 0 |
| drop in | 2 | 0 |
| on behalf of | 2 | 0 |
| tradition | 1 | 0 |
| interpersonal | 1 | 0 |
| socialise | 1 | 0 |
| visits | 1 | 0 |
| visit | 1 | 0 |
| calls on | 1 | 0 |

Table S5. Words in The Drinker Corpus belonging to E1 Emotional actions, states and processes.

| **Word** | **Raw freq.** | **Rel freq.** |
| --- | --- | --- |
| feel | 28 | 0.08 |
| mood | 24 | 0.06 |
| emotional | 11 | 0.03 |
| emotions | 4 | 0.01 |
| flushed | 2 | 0.01 |
| emotionally | 2 | 0.01 |
| gut feeling | 1 | 0 |
| feeling | 1 | 0 |
| feels | 1 | 0 |
| moody | 1 | 0 |
